# Phytochemical Profile and Biological Activities of the Endemic *Thymbra nabateorum* Occurring in AlUla County, NW Saudi Arabia

**DOI:** 10.3390/molecules30030657

**Published:** 2025-02-01

**Authors:** Mohamed Djamel Miara, Sami Youssef, Yassine Fendane, Hassan Boukcim, Sami D. Almalki, Shauna K. Rees, Benjamin P. Y.-H. Lee, Abdalsamad Aldabaa, Hamdi Bendif, Ahmed H. Mohamed

**Affiliations:** 1 Department of Research and Development, Valorhiz SAS, 34090 Montpellier, France; sami.youssef@valorhiz.com (S.Y.); yassine.fendane@valorhiz.com (Y.F.); hassan.boukcim@valorhiz.com (H.B.); 2 Department of Ecology and Biotechnology, Faculty of Nature and Life Science, IBN Khaldoun University of Tiaret, Tiaret 14000, Algeria; 3 Wildlife and Natural Heritage, Royal Commission for AlUla, AlUla 43544, Saudi Arabia; s.almalki@rcu.gov.sa (S.D.A.); s.rees@rcu.gov.sa (S.K.R.); b.yihann@rcu.gov.sa (B.P.Y.-H.L.); a.aldabaa@rcu.gov.sa (A.A.); ah.mohamed@rcu.gov.sa (A.H.M.); 4 Plant Ecology and Rangeland Management, Desert Research Center, Cairo 11753, Egypt; 5 Biology Department, College of Science, Imam Mohamad Ibn Saud Islamic University (IMSIU), Riyadh 11623, Saudi Arabia; hlbendif@imamu.edu.sa; 6 Pedology Department, Desert Research Center, Cairo 11753, Egypt

**Keywords:** *Thymbra*, phytochemical composition, enzyme inhibition, antioxidant activity, antidiabetic effects, natural products, cytotoxic effects

## Abstract

*Thymbra nabateorum*, a plant species from the Lamiaceae family, is endemic to the Nabatian region, which spans southern Jordan and northwestern Saudi Arabia. It is renowned for its traditional uses and rich phytochemical profile. This study aims to examine the phytochemical composition of *T. nabateorum* and evaluate its biological activities, including antioxidant capacity, cytotoxic effects on cancer cell lines, and enzyme inhibition relevant to diabetes and neurodegenerative diseases. The essential oil (EO) and methanol extract of *T. nabateorum* were analyzed using Gas Chromatography–Mass Spectrometry (GC-MS) and High-Performance Liquid Chromatography (HPLC). Antioxidant activity was assessed using the DPPH radical scavenging assay. Cytotoxicity was evaluated against MDA-MB231 and LNCaP cancer cell lines using the MTT assay. Enzyme inhibition assays were conducted to determine the inhibitory effects on α-amylase, α-glucosidase, and butyrylcholinesterase. GC-MS analysis revealed thymol (82.30%) as the major component of the essential oil, while HPLC identified significant phenolic compounds in the methanol extract, including diosmin (118.75 mg/g) and hesperidin (22.18 mg/g). The DPPH assay demonstrated strong antioxidant activity, with the methanol extract showing an IC50 of 11.97 μg/mL for α-amylase and 31.99 μg/mL for α-glucosidase, indicating notable antidiabetic potential. Cytotoxicity tests revealed significant antiproliferative effects against both cancer cell lines, with lower IC50 values compared to standard treatments. *T. nabateorum* exhibits substantial antioxidant, cytotoxic, and enzyme inhibition activities, supporting its traditional medicinal uses. These findings provide a scientific basis for further research into its bioactive compounds and potential applications in modern pharmacology, particularly in developing natural therapeutic agents for oxidative stress-related diseases and cancer.

## 1. Introduction

Natural products are vital in modern strategies for treating diseases, with the potential to significantly contribute to the discovery of new therapeutic agents [1]. Identifying bioactive compounds from natural sources is a complex process involving initial biological investigations of raw materials, followed by phytochemical studies, chromatographic separations, and spectroscopic analyses. These steps are usually followed by comprehensive biological evaluations. Pre-clinical trials and clinical applications have led to the development of numerous clinically used drugs and templates for pharmaceutical formulations [2,3,4].

*Thymbra nabateorum* (Danin & Hedge) Bräuchler, formerly named *Satureja nabateorum Danin & Hedge*, is a perennial herb of the Lamiaceae family, which encompasses over 235 genera and 7000 species globally. This family is well known for its culinary and pharmaceutical importance, owing to its richness in volatile oils and phytochemicals such as polyphenols, flavonoids, and terpenoids [5,6,7]. Plants of this family, including popular species such as thyme, oregano, basil, and rosemary, are widely used in herbal teas, soft drinks, and traditional medicine [8,9]. The genus *Satureja*, including taxa recently ranged in *Thymbra* [10], consists of over 200 species native to the Mediterranean, Middle East, West Asia, and South America. Species of this genus exhibit significant biological activities, including antioxidant, anticancer, antidiabetic, and antimicrobial effects [8,11,12]. *T. nabateorum* has been traditionally used in folk medicine, particularly in arid environments for its purported health benefits, including antimicrobial, anti-inflammatory, and antioxidant properties [8,13]. Indeed, local Bedouins in the Arabian Peninsula used the plant as a cold beverage to alleviate gastrointestinal cramps and treat livestock ailments [14]. Despite these traditional uses, very little scientific literature is available on this plant. In fact, only two studies have been carried out on its chemical composition and biological activities in Palestine [14] and Saudi Arabia [8]. They have shown that its essential oils (EOs) are rich in thymol γ-terpinene and p-cymene, while its extracts have a potent anticancer, antimicrobial, and antioxidant effect. In reality, regardless of these encouraging results, it seems that much potential remains to be discovered by further exploring this plant on the phytochemical level.

The exploration of medicinal plants like *T. nabateorum* is critical due to the increasing demand for natural remedies and the limitations of synthetic drugs [15]. Phytochemical compounds that these valuable plants may contain such as polyphenols, flavonoids, and terpenoids have demonstrated diverse pharmacological activities, including antioxidant, anticancer, and antidiabetic effects [16,17]. Antioxidant activity is particularly relevant as it helps mitigate oxidative stress—a key factor in chronic diseases like diabetes and cancer [18]. Furthermore, the inhibition of metabolic enzymes, such as α-amylase and α-glucosidase could highlight its potential for diabetes management [19].

This study seeks to fill the knowledge gap regarding *T. nabateorum* by analyzing its phytochemical composition and evaluating its biological activities, including antioxidant capacity, cytotoxicity against cancer cell lines, and enzyme inhibition relevant to diabetes.

## 2. Results

### 2.1. Quantitative Analysis of T. nabateorum Methanolic Extract by HPLC-DAD

**Figure 1:**
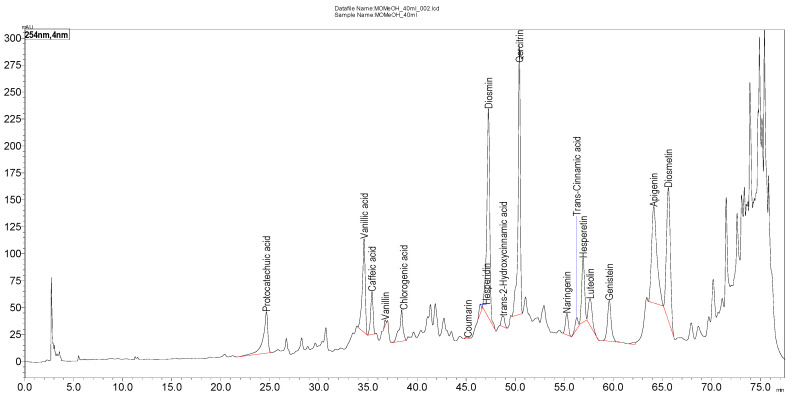
HPLC–DAD chromatogram of *T. nabateorum* methanolic extract at 254 nm (Inertsil ODS-3 column (4 μm, 4 mm × 150 mm). Mobile phase: 0.1% acetic acid–methanol (gradient elution). Flow rate: 1 mL/min. Diode array detection: 254 nm.

Table 1 shows the phenolic composition of the *T. nabateorum* methanol extract, quantified using HPLC-DAD (High-Performance Liquid Chromatography with Diode Array Detection). The table lists various phenolic compounds along with their respective retention times (RT) and concentrations expressed in milligrams per gram of extract (mg/g). Figure 1 illustrates the HPLC-DAD chromatogram of the *T. nabateorum* methanol extract, providing a visual representation of the separation and detection of phenolic compounds.

**Figure 2:**
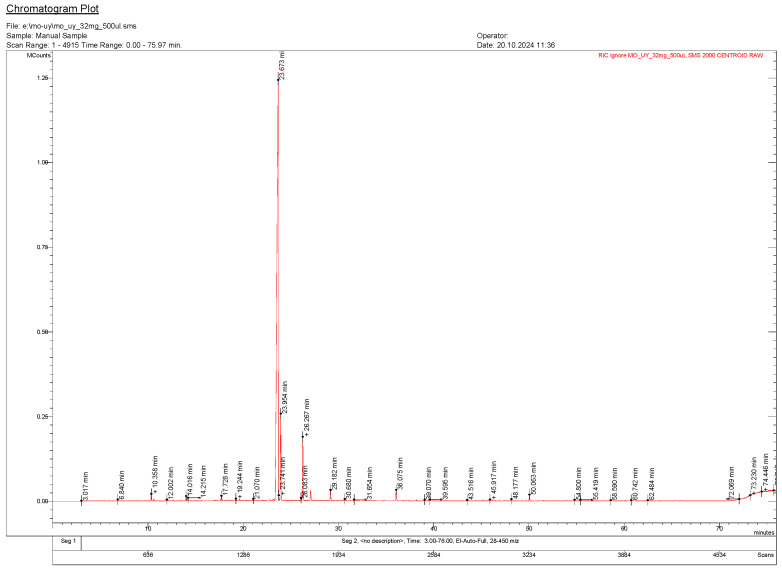
GC-MS chromatogram of *T. nabateorum* essential oil.

The phenolic composition of *T. nabateorum* methanol extract, as outlined in Table 1, reveals a diverse array of bioactive compounds. The extract is particularly rich in diosmin (118.75 mg/g), the most abundant compound, known for its antioxidant and anti-inflammatory properties that support cardiovascular health and venous function. Hesperetin (26.34 mg/g) and hesperidin (22.18 mg/g) further enhance the extract’s therapeutic profile through their potent antioxidant and anti-inflammatory activities. Other notable compounds, such as quercitrin (6.77 mg/g), vanillic acid (2.53 mg/g), and caffeic acid (1.33 mg/g), contribute additional antioxidative capabilities, while trace amounts of coumarin may synergistically enhance the bioactivity of the extract. The description of Figure 2 provides details about the HPLC-DAD (High-Performance Liquid Chromatography with Diode Array Detection) chromatogram for the methanol extract of *T. nabateorum*. The richness and diversity of phenolic compounds suggest that *T. nabateorum* could serve as a valuable source of natural antioxidants and anti-inflammatory agents, with applications in functional foods, dietary supplements, and herbal medicine. Moreover, its phenolic profile opens avenues for further research into its efficacy against conditions linked to oxidative stress, inflammation, and cardiovascular health. This positions *T. nabateorum* as a promising candidate for future health-related studies and applications.

### 2.2. Essential Oil Composition of Thymbra nabateorum by GC-MS Analysis

Table 2 presents the results of a Gas Chromatography–Mass Spectrometry (GC-MS) analysis of the essential oil extracted from *T. nabateorum*.

The GC-MS analysis of *T. nabateorum* essential oil identified a total of 25 compounds, representing the majority of the oil’s composition, with three peaks remaining unidentified (accounting for 0.48%) (Figure 2). The oil is dominated by monoterpenes and monoterpenoids (95.90%), with thymol as the most abundant compound (82.30%), followed by carvacrol (6.40%) and thymol acetate (4.91%). Other groups include sesquiterpenes and sesquiterpenoids (1.89%), with β-caryophyllene (0.84%) and caryophyllene oxide (0.93%) as notable components, as well as aliphatic hydrocarbons and alcohols (0.81%), oxygenated compounds (0.69%) and other hydrocarbons (0.25%). Despite their low concentrations, trace compounds like α-terpinene (0.08%) and hexahydrofarnesyl acetone (0.07%). The high number of identified compounds underscores the oil’s chemical complexity.

### 2.3. Fatty Acid and Volatile Composition

**Figure 3:**
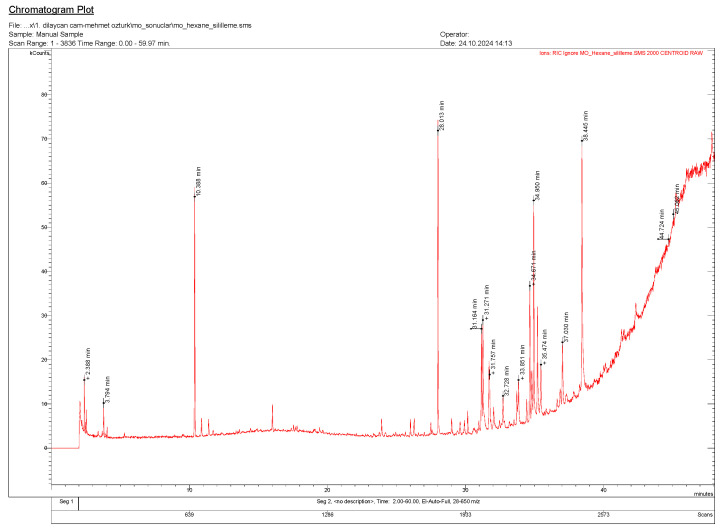
GC-MS chromatogram of *T. nabateorum* silylated hexane extract.

The GC-MS analysis of the hexane extract of *T. nabateorum* (Table 3, Figure 3) reveals a detailed composition of fatty acids, highlighting a significant dominance of saturated fatty acids (SFAs) at 49.82%, compared to unsaturated fatty acids (UFAs) at 9.87%. Key SFAs include docosanoic acid (C22:0) at 17.43%, palmitic acid (C16:0) at 14.72%, and eicosanoic acid (C20:0) at 6.13%, indicating a stable profile less prone to oxidation. The UFAs, comprising linoleic acid (C18:2) at 4.46% and oleic acid (C18:1) at 5.41%, contribute potential health benefits including anti-inflammatory properties, though their proportion is notably lower. The presence of bioactive compounds such as thymol (10.21%) further enhances the extract’s therapeutic potential, suggesting applications in health and industry. This composition, dominated by SFAs, may also provide enhanced stability and shelf life, warranting further exploration of its biological and functional properties.

### 2.4. Mineral Content

The mineral content analysis of *T. nabateorum* compared with NIST-CRM 1203 drinking water highlights key differences in composition (Table 4). While the plant shows low levels of phosphorus (0.18 mg/kg), potassium (1.85 mg/kg), calcium (3.25 mg/kg), magnesium (0.36 mg/kg), copper (8.23 mg/kg), manganese (41.28 mg/kg), zinc (34.17 mg/kg), and boron (22.14 mg/kg), its iron content (755.22 mg/kg) is notably higher, suggesting it may serve as a rich source of iron. High recovery percentages for all measured minerals confirm the accuracy of the analytical methods.

### 2.5. Biological Activities

#### 2.5.1. Antioxidant Properties

The antioxidant activities of *T. nabateorum* extracts evaluated using ABTS+, DPPH, and CUPRAC assays highlight the methanolic extract as the most potent, with low IC50 values (4.02 μg/mL for ABTS+, 9.67 μg/mL for DPPH) and strong CUPRAC activity (A0.5 = 4.37), indicating high free radical scavenging and oxidant-reducing capacity (Table 5). The essential oil exhibits moderate antioxidant activity (ABTS+ IC50 = 17.55 μg/mL, DPPH IC50 = 13.44 μg/mL), while the hexane extract shows the weakest performance, with significantly higher IC50 values (20.78 μg/mL for ABTS+ and 94.44 μg/mL for DPPH). Compared to BHA, a reference antioxidant with robust activity across assays, the methanol extract is competitive, while the hexane extract falls short. These results emphasize the importance of extraction methods in determining antioxidant efficacy, with methanol extraction yielding the highest antioxidant potential, followed by essential oil and hexane extract.

Table 5 demonstrates the correlation between different antioxidant assays for evaluating *T. nabateorum* extracts. The ABTS+, DPPH, and CUPRAC assays show a positive correlation with one another, indicating that extracts with strong antioxidant activity in one assay generally exhibit similar effectiveness in the others. This consistency suggests that these assays are reliable and complementary in assessing the radical-scavenging and oxidant-reducing capacities of the extracts. Overall, the results underscore the importance of multiple assays to provide a comprehensive assessment of antioxidant activity, particularly when certain tests lack complete data.

#### 2.5.2. Anticholinesterase Activity

The enzyme inhibition activities of *T. nabateorum* extracts demonstrate varying levels of potential for health-related applications (Table 6). The methanol extract exhibits the most promising results, with strong antidiabetic activity, particularly against α-amylase (IC50 = 11.97 μg/mL), moderate tyrosinase inhibition (12.81% at 100 μg/mL), and some urease inhibition (10.51% at 50 μg/mL). The hexane extract shows moderate antidiabetic activity, with relatively weaker effects on α-amylase and negligible BChE inhibition, alongside moderate tyrosinase (13.28%) and urease inhibition (17.23%). The essential oil displays moderate tyrosinase (14.26%) and urease (18.13%) inhibition but lacks data on antidiabetic activities. Reference compounds like galantamine, kojic acid, and acarbose highlight the extracts’ potential but underscore that their activity levels are generally lower. Overall, the methanol extract stands out for its strong antidiabetic potential, while all extracts exhibit some level of tyrosinase and urease inhibition, suggesting potential applications in managing diabetes, infections, and skin-related conditions.

#### 2.5.3. Cytotoxicity Analysis of *T. nabateorum* Extracts on Cancer Cell Lines

**Figure 4:**
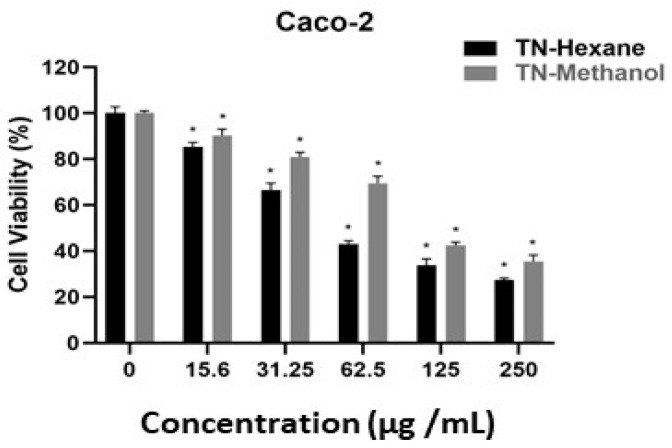
Cytotoxic effects of extracts on Caco-2 cell lines. * Asterisks signify statistically significant differences between the values (*p* < 0.05).

**Figure 5:**
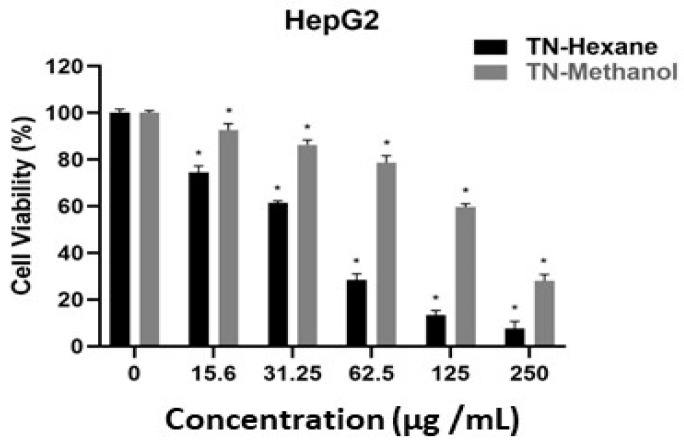
Cytotoxic effects of extracts on HepG2 cell lines. * Asterisks signify statistically significant differences between the values (*p* < 0.05).

**Figure 6:**
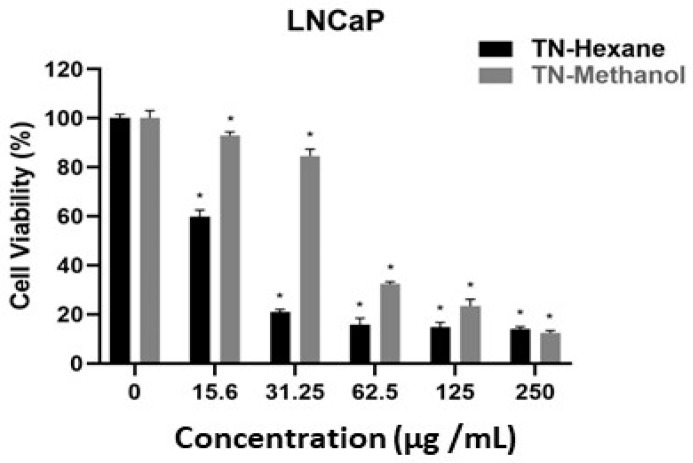
Cytotoxic effects of extracts on LNCaP cell lines. * Asterisks signify statistically significant differences between the values (*p* < 0.05).

**Figure 7:**
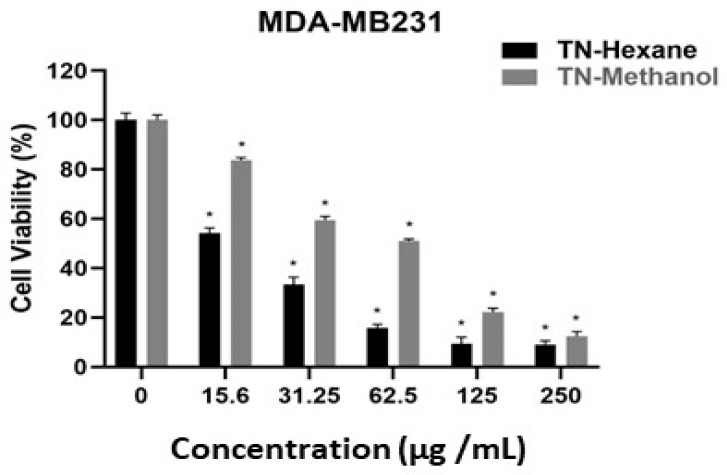
Cytotoxic effects of extracts on MDA-MB231 cell lines. * Asterisks signify statistically significant differences between the values (*p* < 0.05).

**Figure 8:**
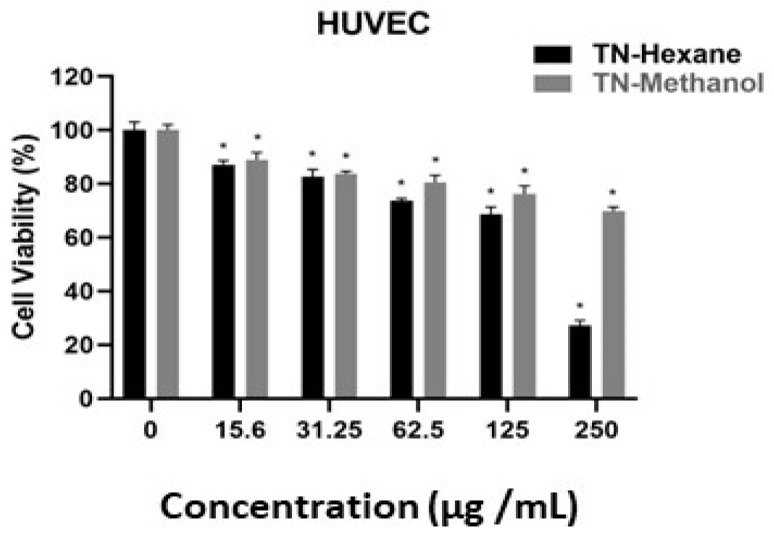
Comparative cytotoxicity of extracts on HUVEC cell lines. * Asterisks signify statistically significant differences between the values (*p* < 0.05).

Table 7 provides EC50 values, indicating the concentration of extract required to inhibit cell growth by 50%. Lower EC50 values suggest higher potency. TN-Hexane extract demonstrates greater cytotoxicity than TN-Methanol, with the lowest EC50 values observed in MDA-MB231 (21.20 µg/mL) and LNCaP (19.60 µg/mL) cancer cell lines. Both extracts show notably higher EC50 values for HUVEC normal endothelial cells (e.g., 190.79 µg/mL for TN-Hexane), suggesting selectivity toward cancer cells. These findings highlight the therapeutic potential of TN-Hexane for selectively targeting cancer cells while sparing normal cells. While Figure 4, Figure 5, Figure 6, Figure 7 and Figure 8 illustrate the cytotoxic effects of *T. nabateorum* extracts, TN-Hexane consistently demonstrates higher potency, reducing cell viability in MDA-MB231, LNCaP, Caco-2, and HepG2 cancer cell lines. Conversely, HUVEC cells show lower sensitivity, underscoring the extracts’ selectivity.

## 3. Discussion

Our study, based on GC-MS and HPLC analysis of *T. nabateorum*, identified thymol as the predominant compound, constituting 82.30% of the essential oil composition. Other significant compounds have been found included carvacrol (6.40%) and thymol acetate (4.91%). Furthermore, HPLC analysis of the methanol extract revealed high concentrations of diosmin (118.75 mg/g), hesperetin (26.34 mg/g), and hesperidin (22.18 mg/g), all contributing to the antioxidant and anti-inflammatory properties of the extract. These findings highlight the significant bioactive potential of *T. nabateorum*, especially in therapeutic and antimicrobial applications.

The findings of the current study are consistent with those of Al-Maharik and Jaradat [14], who reported that the EO of *T. nabateorum* contained thymol as the major compound at 46.07%, followed by γ-terpinene (21.15%) and *p*-cymene (15.02%). Their HPLC-based analysis of the phenolic composition revealed notable amounts of vanillic acid (2.53 mg/g) and caffeic acid (1.33 mg/g). Similarly, Khalil et al. [13] highlighted the significant presence of thymol in *Satureja* species, linking it to antimicrobial and antioxidant properties, though their study placed less emphasis on flavonoid content.

Huwaimel et al. [8] expanded on the bioactivity of thymol-rich species, emphasizing the role of this phenolic compound in modulating oxidative stress and microbial activity. Their findings align with the results of this study, underscoring the importance of thymol as a key bioactive constituent. However, this study reveals a significantly higher thymol concentration in *T. nabateorum*, which could suggest a stronger potential for bioactivity compared to the study of Huwaimel et al. [8]. The substantial flavonoid content in *T. nabateorum*, particularly diosmin, hesperetin, and hesperidin, further differentiates it, indicating broader antioxidant and anti-inflammatory applications.

Regarding nutritional and mineral composition, no previous study has provided a comprehensive analysis of the composition of *T. nabateorum*. This research fills a critical gap, revealing a unique profile that distinguishes it from other *Thymbra* species. Interestingly, its notably high iron content (755.22 mg/kg), exceeding the reported range of 102.52–684.0 mg/kg [20,21,22], suggests potential for addressing iron deficiencies. While its potassium content (1.85%) is lower than the range of 15,610–68,250 mg/kg reported in other species [23,24,25,26,27], its calcium levels (3.25%) align with the reported 11,416–29,965 mg/kg range [23,24,25]. Magnesium (0.36%) is lower than the 1923–3598 mg/kg range [20,24,25], but zinc (34.17 mg/L) exceeds the 9.97–557 mg/kg range [20,21,25]. Its copper content (8.23 mg/L) is comparable to the 6.77–136.3 mg/kg range [20,21,24].

Existing studies on other *Thymbra* species report protein content ranging from 12.70% to 19.51%, crude fiber from 8.89% to 30.63%, crude fat from 6.23% to 19.97%, and carbohydrates around 54.61% [23,24,25,26,27], offering a foundation for nutritional comparisons. The distinct mineral and nutritional profile of *T. nabateorum* highlights its potential for dietary and therapeutic applications. Its high iron and zinc content, along with its comparable calcium and copper levels, suggest enhanced bioactivity that could support antioxidant, antimicrobial, and nutraceutical applications, warranting further investigation into its health benefits and functional uses.

The antioxidant activity of *T. nabateorum* (syn. *Satureja nabateorum*) has been demonstrated through both its methanol extract and essential oils. Data from this study on the antioxidant potency of *T. nabateorum* show that the methanol extract exhibited IC50 values of 4.02 μg/mL for ABTS+ and 9.67 μg/mL for DPPH, confirming its strong free radical scavenging activity. The essential oil of *T. nabateorum* showed a comparable DPPH IC50 value of 4.78 μg/mL, which is consistent with the findings of Al-Maharik and Jaradat [14], who reported DPPH IC50 values of 4.78 and 5.37 μg/mL for EOs extracted from air-dried and fresh samples, respectively. These results underscore the significant antioxidant potential of *T. nabateorum* EOs and extracts.

Moreover, the methanol extract yielded the highest antioxidant activity, followed by the essential oil, which is in line with Huwaimel et al. [8], who emphasized the antioxidant efficacy of essential oils in comparison to other extraction methods. In contrast, the hexane extract in this study demonstrated the weakest antioxidant performance (IC50 values of 20.78 μg/mL for ABTS+ and 94.44 μg/mL for DPPH), highlighting the influence of extraction method and solvent choice on the bioactive compound yield and antioxidant activity. These findings align with Huwaimel et al. [8], who reported the varying antioxidant capacities based on extraction solvents.

Comparing *S. nabateorum* to other species within the *Satureja* genus, the results of this study are consistent with previous studies. Elgndi et al. [28] demonstrated significant antioxidant activity in *Satureja montana* EOs, reporting an IC50 value of 5.05 ± 1.52 μg/mL. Additionally, Aghbash et al. [29] found that EOs from *Satureja macrantha* at various growth stages exhibited antioxidant activities ranging from 12.14 ± 0.63 to 21.85 ± 1.02 μg/mL, indicating that *T. nabateorum* possesses superior antioxidant properties. Similarly, Giweli et al. [30] documented that *Satureja thymbra* EOs had a much higher IC50 value of 96.7 μg/mL, making them significantly weaker in antioxidant capacity compared to *T. nabateorum*.

Furthermore, Ghotbabadi et al. [31] assessed the antioxidant capacity of *Satureja sahendica* EOs, finding that the pre-flowering stage exhibited the highest radical scavenging activity with an IC50 value of 7.85 ± 0.06 μg/mL. This result also demonstrates that *T. nabateorum* EOs are more potent in comparison.

The observed antioxidant activity in *T. nabateorum* is primarily attributed to bioactive compounds such as thymol and other phenolics, which are well known for their free radical scavenging properties. This is consistent with the findings of Khalil et al. [13], who emphasized the role of thymol and other phenolic compounds in the antioxidant properties of *Satureja* species. Additionally, the positive correlation observed across multiple antioxidant assays (ABTS+, DPPH, and CUPRAC) validates the reliability of these methods for evaluating antioxidant capacity.

Overall, these findings confirm that *T. nabateorum* exhibits superior antioxidant potential compared to many other species in the *Satureja* genus, both in terms of essential oils and extracts, further emphasizing its value in natural product research and health applications.

This study on *T. nabateorum* reveals strong enzyme inhibition activities, particularly against α-amylase, α-glucosidase, and cholinesterase enzymes. Specifically, the methanol extract showed the most promising results, with an IC50 of 11.97 μg/mL for α-amylase inhibition and 31.99 μg/mL for α-glucosidase inhibition, indicating potential antidiabetic activity. The methanol extract also exhibited moderate inhibition of butyrylcholinesterase (BChE), with an IC50 of 122 μg/mL, suggesting possible applications in Alzheimer’s disease therapy. In addition, the essential oil showed weaker inhibitory effects compared to the methanol extract, with some activity against α-amylase and α-glucosidase. The activity observed in our extracts also aligns with findings reported by Jaradat et al. [32], who documented significant acetylcholinesterase (AChE) and butyrylcholinesterase (BChE) inhibition, with IC50 values of 28.24 μg/mL and 92.31 μg/mL, respectively. Notably, the results of this study demonstrated comparable inhibition levels, indicating a potential role for these extracts as natural alternatives for managing neurodegenerative disorders such as Alzheimer’s disease. This is particularly important given the increasing demand for non-synthetic therapeutic options to address resistance to conventional treatments.

The essential oil also showed some inhibitory activity, though weaker than the methanol extract. In comparison, the study by Jaradat et al. [32] reported that *Satureja* species exhibited IC50 values for α-amylase inhibition ranging from 30 to 70 μg/mL and α-glucosidase inhibition between 50 and 100 μg/mL, which are higher than those observed in our *T. nabateorum* extract. Furthermore, the Jaradat et al. [32] study found that plant extracts, such as those from *Mentha* and *Satureja hortensis*, inhibited acetylcholinesterase (AChE) with IC50 values around 20–70 μg/mL, while this study showed moderate inhibition of butyrylcholinesterase (BChE) in *T. nabateorum* extracts, with an IC50 of 122 μg/mL. These results suggest that *T. nabateorum* promising candidate for further exploration in both diabetes and Alzheimer’s disease treatments.

Cancer remains one of the leading causes of death globally, with chemotherapy often limited by issues such as drug resistance and lack of selectivity for cancer cells [33]. This necessitates the exploration of natural compounds derived from medicinal plants, which show promise in developing novel anticancer treatments [34]. The extracts of *T. nabateorum* exhibit significant cytotoxicity, particularly the TN-Hexane extract, which demonstrated the lowest EC_50_ values for MDA-MB231 (21.20 μg/mL) and LNCaP (19.60 μg/mL) cancer cell lines, indicating its high potency. Additionally, both extracts showed notably higher EC_50_ values for normal endothelial cells (e.g., 190.79 μg/mL for TN-Hexane), highlighting their selectivity for cancer cells. These results align with studies by Al-Maharik and Jaradat [14], which revealed that thymol, c-terpinene, and p-cymene in *T. nabateorum* extracts exerted cytotoxic effects on MCF-7, COLO-205, HeLa, and HepG2 cell lines.

Furthermore, the cytotoxicity of TN-Hexane against various cancer cell lines, including MDA-MB231, Caco-2, and HepG2, underscores its therapeutic potential, as illustrated in Figure 4, Figure 5, Figure 6, Figure 7 and Figure 8 and Table 7. Comparative analyses show that TN-Hexane outperforms TN-Methanol in potency, while its selective cytotoxicity likely stems from active compounds such as thymol and carvacrol, consistent with findings on *Satureja thymbrifolia* [28,35]. These results align with the broader evidence supporting natural products as anticancer agents, including their ability to induce apoptosis and growth arrest in cancer cells without affecting healthy cells [19]. Moreover, the antiproliferative effects of *Satureja* extracts, such as SNS and SNL, against MCF-7, HepG2, Panc-1, and A549 cell lines, further highlight the therapeutic potential of these plants, with SNS showing superior potency [14]. This reinforces the potential of *T. nabateorum* as a promising source for developing targeted, natural cancer therapies with minimal impact on healthy cells.

The bioactivity and chemical composition of *T. nabateorum* are central to its antioxidant properties, which make it a promising candidate for natural product research. The bioactive compounds present in both essential oils and extracts, such as thymol and other phenolic compounds, are well known for their ability to scavenge free radicals, contributing significantly to the observed antioxidant activity. The variation in bioactive compound yield depending on the extraction method—whether through methanol, essential oils, or hexane—further emphasizes the importance of choosing the right extraction technique to maximize antioxidant efficacy. Additionally, the chemical composition of *T. nabateorum* plays a critical role in its potential therapeutic applications. By isolating and characterizing the bioactive compounds, researchers can better understand the mechanisms behind their antioxidant effects, paving the way for targeted health applications. This underscores the importance of studying both the bioactivity and chemical composition to fully harness the benefits of this plant in natural antioxidant therapies.

## 4. Materials and Methods

### 4.1. Chemicals and Reagents

The study utilized several key chemicals and reagents, including dimethyl sulfoxide (DMSO) as a solvent for dissolving extracts, penicillin/streptomycin as an antibiotic solution for cell culture, and fetal bovine serum (FBS) as a supplement for cell culture media. Nutrient media for cell culture were provided by DMEM/RPMI Medium, while MTT Reagent was employed to assess cell viability in the MTT assay. Bioactivity measurements were conducted using a 96-well microplate reader (SpectraMax 340PC384, Molecular Devices, united stat of America (San Jose, CA, USA)), with results analyzed using Softmax PRO v5.2 software. Gas Chromatography–Mass Spectrometry (GC-MS) analyses were performed on a Varian Saturn 2100T coupled with a 3900GC (Santa Clara, CA, USA). Various chemicals, including ammonium acetate, boron trifluoride-methanol complex, copper (II) chloride, ethanol, methanol, and n-hexane, were sourced from E. Merck (Darmstadt, Germany). Additionally, 1,1-diphenyl-2-picrylhydrazyl (DPPH), 2,2′-Azino-bis(3-ethylbenzothiazoline-6-sulfonic acid) diammonium salt (ABTS), 4-N-nitrophenyl-α-D-glucopyranoside (PNPG), 5,5-Dithiobis(2-nitrobenzoic acid) (DTNB), acetylcholinesterase from electric eel (AChE, Type-VI-S, EC 3.1.1.7, 425.84 U/mg, acetylthiocholine iodide acid, butylated hydroxyl anisole (BHA), butyrylcholinesterase from horse serum (BChE, EC 3.1.1.8, 11.4 U/mg, Sigma), butyrylthiocholine chloride, galantamine, urease from *Canavalia ensiformis* (Jack bean) (EC 232-656-0, Type III, powder, 15,000–50,000 units/g solid), thiourea, urea, phenol reagent, alkali reagent, kojic acid, L-DOPA (3,4-Dihydroxy-D-phenylalanine), neocuproin, phosphate buffer, potassium persulfate, tyrosinase from mushroom (EC 232-653-4, 250 KU, ≥1000 U/mg solid, α-amylase, α-glucosidase, and Lugol’s reagent were obtained from Sigma Chemical Co. (Sigma-Aldrich GmbH, Sternheim, Germany). All other chemicals and solvents used were of analytical grade.

### 4.2. Plant Materials

**Figure 9:**
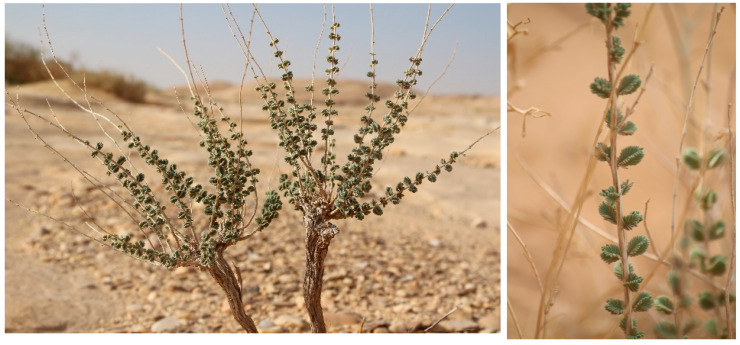
Illustration of *T. nabateorum* in its natural environment.

The aerial part (the stems, leaves, and flowers) of *T. nabateorum* was collected in the Sharaan National Park, AlUla County, Saudi Arabia (Figure 9) in February 2024, at an altitude of 1099 m, with geolocation coordinates 38.45778833° N, 26.972795° E. The available literature [36] was used to identify and confirm the species by Valorhiz botanists (M.D.Miara, S.Y). An archived specimen has been stored in the herbarium of the Royal Commission of AlUla, Saudi Arabia (specimen number: 139,351).

### 4.3. Preparation of Crude Extracts

Before extraction, the aerial parts of the plant were thoroughly cleaned to remove impurities and dried in a well-ventilated, humidity-free environment at room temperature for seven days. During this process, care was taken to avoid direct exposure to sunlight to preserve the plant’s bioactive compounds. Once dried, the aerial parts of the plant were crushed into a fine powder using an electric milling machine, preparing them for extraction. The plant material (100 g) was extracted with n-hexane three times (3 × 500 mL). The residue was then extracted with methanol three times (3 × 500 mL) [37]. The solvent was refreshed at the beginning of each extraction attempt to ensure efficient compound recovery, and the extracts were concentrated by evaporating the solvent under vacuum [37]. All filtrates were stored at 4 °C until further analysis to maintain their stability and prevent degradation of the extracted compounds.

### 4.4. Essential Oil Extraction

The essential oil from the dried aerial parts of *T. nabateorum* (300 g) was obtained through hydrodistillation using a Clevenger-type apparatus [38]. The distillation process was carried out over a period of 4 h, utilizing steam to facilitate the release of volatile compounds. Following distillation, the collected essential oil was dried by passing it over anhydrous magnesium sulfate, which effectively removed any residual moisture. To ensure the oil’s stability and prevent oxidative degradation, it was stored under an inert nitrogen atmosphere in a sealed container until further use.

### 4.5. Preparation of n-Hexane Extract

To analyze the *n*-hexane extract of *T. nabateorum*, 5 mg of the extract was dissolved in 75 μL of pyridine, and 50 μL of bis (trimethysilyl) trifluoroacetamide was added. The tube was placed in an oven to incubate at 60 °C for 20 min. After cooling, the mixture was diluted to 500 μL. Then, 0.2 μL was injected into GC-MS [39].

### 4.6. GC-MS Analysis of n-Hexane Extract and Essential Oil

A Varian Saturn 2100T ion trap analyzer (California, USA). coupled with a non-polar DB-5 capillary column (0.25 mm ID, 0.25 µm film thickness, 30 m length) was used for GC-MS analysis. Helium was used as the carrier gas, with a flow rate of 1.3 mL/min. The temperature program for silylated *n*-hexane extract column temperature began at 100 °C for 5 min, then ramped to 300 °C at a rate of 5 °C/min, where it was maintained for 7 min and increased to 320 °C at a rate of 20 °C/min, where it was maintained for 7 min. For the temperature program for the essential oil, the column temperature began at 60 °C for 5 min, then increased to 240 °C at a rate of 3 °C/min, where it was maintained for 10 min and increased to 320 °C at a rate of 25 °C/min, where it was maintained for 5 min. Electron ionization at 70 eV was applied. The injector, transfer line, manifold, and ion trap temperatures were set at 250 °C, 180 °C, 120 °C, and 150 °C, respectively. The compounds were identified by comparing the obtained spectra with the NIST/Wiley 2014 mass spectral library and hydrocarbon (C_7_–C_30_) standards [38,39].

### 4.7. High-Performance Liquid Chromatography (HPLC-DAD) Analysis

The phenolic components in the methanolic extract of *T. nabateorum* were analyzed using a Shimadzu reversed-phase HPLC-DAD system (Shimadzu Cooperative, Kyoto, Japan) based on a validated method involving 43 standard compounds [40]. The separation was carried out on an Inertsil ODS-3 column (4 μm film thickness, 150 mm × 4.0 mm) coupled with an Inertsil ODS-3 guard column, with the column temperature maintained at 40 °C. Stock solutions of the extract (8 mg/mL) were prepared in a methanol/water (80/20, *v*/*v*) mixture and pre-filtered through disposable 0.45 µm LC disk filters (Agilent, Santa Clara, CA, USA). The mobile phase consisted of 0.5% acetic acid in water (A) and methanol (B). A gradient elution program was applied over 40 min: 0–0.01 min (0–20% B), 0.01–2 min (20–60% B), 2–15 min (60–80% B), 15–30 min (100% B), 30–35 min (100–10% B), and 35–40 min (10–0% B). The flow rate was set at 1.5 mL/min, and 20 µL of the sample was injected for each analysis. Identification of phenolic compounds was performed using a diode array detector (DAD) in the wavelength range of 230–350 nm, referencing the UV data and retention times of commercial standards. All samples and standards were filtered using Agilent 0.45 µm filters. Three replicates were performed for each analysis and the phenolic compounds were quantified and reported as mg/g of dry weight of extract after comparison of retention times.

### 4.8. Mineral Analyses

The sample preparation followed a multi-step process based on the method outlined by Cicero et al. [41], with a few modifications. Initially, the collected samples of *T. nabateorum* were cleaned, chopped, and dried for 24 h at 105 °C using a Nüve oven (Istanbul, Turkey). After drying, the samples were homogenized with an IKA homogenizer (Staufen, Germany) and sieved through a 10-mesh screen to obtain particles of approximately 1600 µm in size. These particles were stored in sanitized polyethylene containers. Deionized water, with a conductivity of 18.2 MΩ·cm^−1^, was used throughout the process, sourced from a Milli-Q^®^ system (Human Power I Plus, Sejong-si, Republic of Korea). All equipment was disinfected with a 10% nitric acid and deionized water solution to prevent contamination. For digestion, 0.5 g of the dried plant material was mixed with 6 mL of 65% nitric acid (HNO_3_) and 2 mL of 30% hydrogen peroxide (H_2_O_2_), using a CEM Mars 5 microwave-sealed system (Matthews, NC, USA). The digestion process was performed under the following conditions: 1000 W power, a temperature range of 150–200 °C, a 20 min ramp, and a 2 min hold at full power. After digestion, the samples were cooled, filtered, diluted with 100 mL of ultra-pure water, and stored at 4 °C. The mineral content was analyzed using Inductively Coupled Plasma Optical Emission Spectroscopy (ICP-OES), Mineral concentrations on a dry-weight basis were also analyzed using an Agilent 7700× Inductively Coupled Plasma Mass Spectrometry (ICP-MS). Blank samples were included to monitor potential contamination. Detailed operating conditions for the ICP-MS analysis are outlined in Table 8.

### 4.9. Antioxidant Activity

The free radical scavenging activities of *T. nabateorum* extracts and oil were evaluated using four different assays: DPPH, ABTS, and CUPRAC.

The DPPH radical-scavenging capacity was assessed following the method by Blois [42], where a 0.1 mM DPPH solution was mixed with varying concentrations of extract, and absorbance was measured at 517 nm after 30 min of incubation in the dark. The scavenging activity was calculated using the formula:

Scavenging activity % = ([Abs_control_ − Abs_sample_]/Abs_control_) × 100, with results reported as IC_50_ (g/mL). For the ABTS cation radical assay [43], the ABTS solution was prepared by reacting potassium persulfate with ABTS, incubating it in the dark for 12 h, and then measuring the absorbance at 734 nm after dilution. The ABTS scavenging effect was calculated similarly, with IC_50_ values used to determine the sample concentration for 50% inhibition. The CUPRAC assay, adapted from Yigitkan et al. [44], involved the addition of sample solutions to a mixture containing ammonium acetate buffer, neocuproine, and Cu (II) solution, followed by incubation and measurement at 450 nm. Results were expressed as A_0.5_ values (g/mL), representing the concentration needed to achieve an absorbance of 0.50. In all assays, butylated hydroxylanisole (BHA) was used as the reference antioxidant for comparison.

### 4.10. Enzyme Inhibition Activity

The enzyme inhibition activities of *T. nabateorum* extracts were evaluated to understand their potential to modulate key biological processes through the inhibition of specific enzymes. The study assessed the extracts’ anticholinesterase activity by targeting acetylcholinesterase (AChE) and butyrylcholinesterase (BChE), enzymes associated with neurological disorders such as Alzheimer’s disease. Additionally, the extracts’ inhibitory effects on tyrosinase, an enzyme linked to melanin production and hyperpigmentation, and urease, an enzyme implicated in infections and gastric conditions, were determined. The antidiabetic potential was further explored by measuring the IC_50_ values of the extracts against α-amylase and α-glucosidase, enzymes critical in carbohydrate metabolism and blood sugar regulation. These comprehensive evaluations highlight the therapeutic promise of *T. nabateorum* extracts for managing various health conditions.

The enzyme inhibitory activity of methanolic and *n*-hexane extracts and oil of *T. nabateorum* against acetylcholinesterase (AChE) and butyrylcholinesterase (BChE) was assessed using the method of Ellman et al. [45], with modifications. For the assay, 150 μL of phosphate buffer was mixed with 10 μL of sample solution dissolved in ethanol, followed by adding 20 μL of either AChE or BChE enzyme solution. The mixture was incubated at 25 °C for 15 min. Subsequently, 10 μL of 0.5 mM DTNB was added. The reaction was initiated by adding 10 μL of the substrate acetylthiocholine iodide (0.71 mM) or butyrylthiocholine chloride (0.2 mM). The hydrolysis of these substrates was monitored by measuring the increase in absorbance at 412 nm every 5 min over a 15 min period using a 96-well microplate reader. Galantamine, a known cholinesterase inhibitor, was used as the positive control at concentrations equivalent to the test samples. Each assay was performed in triplicate for accuracy.

The percentage inhibition of AChE and BChE was calculated using the following formula:

Inhibition (%) = [(enzyme activity without extract − enzyme activity with extract)/enzyme activity without extract] × 100

This method provided insights into the inhibitory potential of the extracts, contributing to understanding their possible therapeutic applications for conditions such as Alzheimer’s disease.

The antidiabetic potential of *T. nabateorum* methanolic and petroleum ether extracts and oil were evaluated through their inhibitory activities against α-amylase and α-glucosidase, enzymes critical in carbohydrate metabolism. Both assays were performed spectrophotometrically using a 96-well microplate reader, with Softmax PRO v5.2 software employed for data analysis. Acarbose, a standard antidiabetic agent, was used as a positive control.

The method described by Quan et al. [46] was followed for α-amylase inhibitory activity with minor adjustments to incubation times, reagent volumes, and sample concentrations. A mixture of 50 µL of α-amylase solution and 25 µL of sample solution in 20 mM phosphate buffer (pH 6.9, with 6 mM NaCl) was pre-incubated at 37 °C for 10 min. Following this, 50 µL of 0.05% starch solution was added, and the reaction mixture was incubated again at 37 °C for another 10 min. The reaction was terminated by adding 100 µL of Lugol’s iodine solution and 25 µL of 0.1 M HCl. Absorbance was measured at 565 nm using the microplate reader. The concentration of extract required to achieve 50% inhibition (IC_50_) was calculated from the dose–response curve.

The α-glucosidase assay followed the method of Kim et al. [47], with minor modifications to reagent volumes and incubation periods. The reaction mixture contained 25 µL of 4-nitrophenyl-α-D-glucopyranoside (PNPG), 50 µL of 0.01 M phosphate buffer (pH 6.9), 10 µL of sample solution in phosphate buffer, and 25 µL of 0.01 M phosphate buffer (pH 6.0) containing α-glucosidase. After incubating at 37 °C for 20 min, 90 µL of 0.1 M Na_2_CO_3_ solution was added to terminate the reaction. Absorbance was measured at 400 nm. The IC_50_ values were calculated from the dose–response curve of inhibitory activity versus sample concentration.

These assays provide insights into the potential of *T. nabateorum* extracts as natural inhibitors of carbohydrate-digesting enzymes, contributing to their potential application in managing postprandial hyperglycemia and diabetes.

### 4.11. Urease Inhibitory Activity

The anti-urease activity of the extracts was evaluated using the indophenol method [48], with urease. The urease enzyme solution was prepared in 100 mM sodium phosphate buffer (pH 8.2). A mixture of 25 μL of urease enzyme, 50 μL of 100 mM urea, and 10 μL of the extract was incubated at 30 °C for 15 min. Following this, 45 μL of 1% (*w*/*v*) phenol reagent and 70 μL of 0.005% (*w*/*v*) alkali reagent were added to initiate the reaction. After 50 min of incubation, the absorbance was measured at 630 nm using a microplate reader. Thiourea was used as a reference compound for comparison [49].

#### Tyrosinase Inhibitory Activity

Tyrosinase inhibitory activity was determined using a spectrophotometric method with mushroom tyrosinase enzyme, as described by Masuda et al. [50]. L-Dopa served as the substrate, and kojic acid was used as the reference compound. The specific details of the procedure included preparing reaction mixtures with extracts, tyrosinase enzyme, and substrate, followed by incubation and measurement of absorbance changes to evaluate enzyme inhibition.

These assays highlight the potential of the extracts in inhibiting urease and tyrosinase, enzymes associated with conditions such as ulcers, hyperpigmentation, and other metabolic dysfunctions.

### 4.12. Cell Culture and Cytotoxicity Analysis

Human hepatocellular carcinoma (HepG2), colorectal adenocarcinoma (Caco-2), prostate adenocarcinoma (LNCaP), breast adenocarcinoma (MDA-MB231), and human umbilical vein endothelial (HUVEC) cell lines were purchased from the European Collection of Cell Cultures (ECACC). The cell lines were cultured for the study in DMEM or RPMI medium supplemented with 1% penicillin/streptomycin and 10% fetal bovine serum (FBS) under conditions of 37 °C, 5% CO_2_, and 95% humidity. When the cells reached 80–90% confluency, they were harvested using Trypsin-EDTA enzyme, centrifuged at 2000 rpm for 5 min, and subcultured as described previously [51]. The cytotoxic effects of the extracts used in the study were determined using the MTT [3-(4,5-dimethylthiazol-2-yl)-2,5-diphenyltetrazolium bromide] method as described previously [38]. The extracts (hexane and Methanol) were dissolved in dimethyl sulfoxide (DMSO). Briefly, the cell lines were seeded at a density of 2 × 10³ cell/well in 96-well plates and incubated overnight. The cells were then incubated with different concentrations of the compounds (15.6; 31.25; 62.5 and 125 µg/mL) for 24 h. Subsequently, the medium was removed, and 100 µL of fresh medium and 10 µL of MTT solution (5 mg/mL) were added to each well and incubated at 37 °C for 3–4 h. To dissolve the formed formazan crystals, 50 µL of DMSO was added, and the plates were incubated for 4 h. The results were measured at 590 nm using a microplate reader (Epoch, BioTek, Winooski, VT, USA). All experiments were conducted in triplicate. Cell viability was calculated, as a percentage of the control. The obtained data were analyzed to calculate EC50 values using GraphPad Prism (v.9).

### 4.13. Statistical Analyses

For all biological tests, the results were expressed as the mean ± standard error meaning (SEM), calculated from three independent replicates. Statistical analyses were performed using a one-way analysis of variance (ANOVA) to identify overall differences among groups. Statistical significance was considered at a threshold of *p* < 0.05 for all assessments, ensuring a reliable interpretation of the data.

## 5. Conclusions

The findings of this study underscore the significant potential of *T. nabateorum* as a source of bioactive compounds with therapeutic applications. The essential oil extracted from this plant identified thymol as the predominant compound, constituting 82.30% of the total composition. This high concentration of thymol suggests its critical role in the biological activity and therapeutic properties of the essential oil. Other notable compounds, such as carvacrol (6.40%) and thymol acetate (4.91%), also contribute to the oil’s efficacy, indicating a complex interplay of various constituents that enhance its overall bioactivity. The quantitative analysis of the methanol extract identified key compounds such as hesperidin (22.18 mg/g) and vanillic acid (2.53 mg/g). These phenolic compounds are known for their antioxidant properties, suggesting that *T. nabateorum* may offer health benefits beyond its essential oil. The biological activities of extracts revealed their significant therapeutic potential, as evidenced by several key results, the antioxidant activities are particularly noteworthy, demonstrating its potential as a natural source of bioactive compounds that combat oxidative stress. The extracts exhibited strong radical-scavenging capabilities, effectively neutralizing free radicals and thereby reducing the risk of oxidative damage associated with various chronic diseases. Quantitative assessments revealed impressive results in multiple antioxidant assays, an IC50 value of 4.02 μg/mL in the ABTS assay, IC50 value of 9.67 μg/mL in the DPPH assay, and a CUPRAC value of 4.37 μg/mL, indicating a robust capacity to inhibit lipid peroxidation and protect cellular integrity; the methanol extract demonstrated promising antidiabetic potential through significant inhibition of α-amylase; cytotoxicity assays indicated strong antiproliferative activity against cancer cell lines, with EC50 values of 21.20 μg/mL for MDA-MB231 and 19.60 μg/mL for LNCaP. These findings collectively underscore the multifaceted biological activities of *T. nabateorum*, highlighting its potential for further exploration and development in modern pharmacology and natural product-based therapies. Looking forward, further research is warranted to explore the synergistic effects of the various compounds present in *T. nabateorum*. Investigating the mechanisms of action of thymol and other constituents could provide deeper insights into their therapeutic potential. Additionally, studies focusing on the extraction methods, formulation development, and in vivo efficacy of the essential oil and methanol extract could pave the way for new applications in health and wellness. Moreover, the ecological and sustainable harvesting of *T. nabateorum* should be considered to ensure the conservation of this valuable plant species. As consumer interest in natural and organic products continues to grow, *T. nabateorum* presents a promising avenue for the development of innovative health products that align with modern trends towards sustainability and natural remedies.

## Figures and Tables

**Table 1:** Phenolic composition of the *T. nabateorum* methanol extracted by HPLC-DAD.

N	Phenolic Compounds	RT (min)	mg/g Extract
**1.**	Protocatechuic acid	24.625	1.50
**2.**	Vanillic acid	34.758	2.53
**3.**	Caffeic acid	35.28	1.33
**4.**	Vanillin	36.915	1.55
**5.**	5-Caffeoylquinic acid	38.422	1.50
**6.**	Coumarin	45.178	tr
**7.**	Hesperidin	46.442	22.18
**8.**	Diosmin	47.274	118.75
**9.**	trans-2-Hydroxycinnamic acid	48.243	0.85
**10.**	Quercitrin	50.427	6.77
**11.**	Naringenin	55.518	4.33
**12.**	trans-Cinnamic acid	56.203	0.21
**13.**	Hesperetin	57.47	26.34
**14.**	Genistein	57.739	0.89
**15.**	Luteolin	57.872	0.94
**16.**	Apigenin	64.071	6.07
**17.**	Diosmetin	65.611	4.30

tr: trace amount.

**Table 2:** GC-MS result of *T. nabateorum* essential oil.

Peaks	RI ^a^	Compound Name	Area	%
**1**	920	1-Butoxy-2-propanol	15,228	0.10
**2**	1048	p-Cymene	69,905	0.45
**3**	1050	trans-2-Octenal	21,940	0.14
**4**	1056	α-Terpinene	12,415	0.08
**5**	1100	Linalool	50,445	0.33
**6**	1105	(Z)-2-Nonen-1-ol	32,011	0.21
**7**	1180	4-Terpineol	87,693	0.57
**8**	1190	Dodecene	25,800	0.17
**9**	1200	Dodecane	18,199	0.12
**10**	1236	Thymol methyl ether	16,188	0.10
**11**	1277	Thymol	12,700,000	82.30
**12**	1295	Carvacrol	989,071	6.40
**13**		Unidentified	33,895	0.22
**14**	1352	Thymol acetate	759,626	4.91
**15**	1370	Carvacrol acetate	118,113	0.76
**16**	1415	β-Caryophyllene	129,473	0.84
**17**	1430	Nerylacetone	24,381	0.16
**18**		Unidentified	15,002	0.10
**19**	1570	Caryophyllene oxide	143,213	0.93
**20**	1830	Eudesma-5,11(13)-dien-8,12-olide	22,161	0.14
**21**	1833	Hexahydrofarnesyl acetone	10,678	0.07
**22**		Unidentified	24,330	0.16
**23**	1948	Bianisal	84,441	0.55
**24**	1997	Eicosene	13,606	0.09
**25**	2005	Manoyl oxide	17,916	0.12
Monoterpenes and monoterpenoids				95.90%
Sesquiterpenes and sesquiterpenoids				1.89%
Aliphatic hydrocarbons and alcohols				0.81%
Other oxygenated compounds				0.69%
Other hydrocarbons				0.25%

^a^ Retention index on DB-5 fused silica column (30 m × 0.25 mm ID, 0.25 µm film thickness).

**Table 3:** GC-MS result of *T. nabateorum* silylated hexane extract.

No	RT	RI ^a^	Compound Name	Area	%
**1**	10.388	1310	Thymol	142,145	10.21
**2**	28.013	2043	Palmitic acid (C16:0)	204,806	14.72
**3**	31.164	2203	Linoleic acid (C18:2)	62,042	4.46
**4**	31.271	2208	Oleic acid (C18:1)	75,333	5.41
**5**	31.711	2239	Stearic acid (C18:0)	69,006	4.96
**6**	32.048		Unidentified	17,067	1.23
**7**	32.728		Unidentified	29,084	2.09
**8**	33.725	2340	Nonadecanoic acid (C19:0)	22,593	1.62
**9**	33.851		Unidentified diterpenoid (C20H30O)	43,956	3.16
**10**	34.671		Unidentified	103,015	7.40
**11**	34.805		Unidentified	47,321	3.40
**12**	34.95		Unidentified	162,682	11.69
**13**	35.22	2416	Eicosanoic acid (C20:0)	85,262	6.13
**14**	35.474	2440	Abietic acid	29,646	2.13
**15**	37.03	2530	Heneicosanoic acid (C21:0)	55,127	3.96
**16**	38.445	2616	Docosanoic acid (C22:0)	242,554	17.43
			Unidentified %		28.97
			Identified %		71.03
**Total**					88.66%
Total saturated fatty acids					49.82%
Total unsaturated fatty acids					9.87%
Others					28.97%
Saturation/unsaturation ratio					5.04
Linoleic acid/oleic acid ratio					0.82

^a^ Retention index on DB-5 fused silica column (30 m × 0.25 mm ID, 0.25 µm film thickness.); RT: retention time; RI: retention index on Rxi-5Sil MS (Restek) (Izmir, Turkey) fused silica non-polar capillary column (30 m × 0.25 mm ID, film thickness 0.25 μm).

**Table 4:** Mineral contents of the *T. nabateorum* and NIST-CRM 1203 drinking water (mg/kg).

Mineral Contents	*T. nabateorum*	Certified and Experimental Values of Studied Metals in NIST-CRM 1203 Drinking Water (mg/kg)		
		Certified Value (mg/kg)	Experimental Value ± S.D. (mg/kg) ^a^	Recovery Value (%)
Phosphorus (%)	0.18 ± 0.001 *	-	-	-
Potassium (%)	1.85 ± 0.05 *	-	-	-
Calcium (%)	3.25 ± 0.02	99.78 ± 0.50	100.42 ± 0.95	100.64
Magnesium (%)	0.36 ± 0.01 *	99.77 ± 0.50	100.68 ± 1.02	100.23
Iron (mg L^−1^)	755.22 ± 11.98 *	200.3 ± 1.0	199.89 ± 2.05	99.94
Copper (mg L^−1^)	8.23 ± 0.04	2000 ± 10	202.9 ± 0.12	101.45
Manganese (mg L^−1^) *	41.28 ± 1.11	50.17 ± 0.25	50.02 ± 0.75	99.67
Zinc (mg L^−1^) *	34.17 ± 0.76	1000 ± 5	1003.1 ± 7.8	102.59
Boron (mg L^−1^) *	22.14 ± 0.33	-	-	-

* Values expressed herein are mean ± SEM of three parallel measurements *p* < 0.05. ^a^ Ten times dilution of certified NIST-CRM 1203 drinking water.

**Table 5:** Antioxidant activities of *T. nabateorum* extracts.

Sample	Antioxidant Activity		
	ABTS+ Assay	DPPH Assay	CUPRAC
	IC_50_ µg/mL		(A_0.5_ µg/mL)
Methanol extract	4.02 ± 0.13	9.67 ± 0.03	4.37 ± 0.01
Hexane extract	20.78 ± 0.67	94.44 ± 0.47	47.76 ± 0.02
Essential oil	17.55 ± 1.16	13.44 ± 1.60	NA
BHA	2.76 ± 0.27	5.80 ± 0.35	5.91 ± 0.00

BHA, 2-tert-Butyl-4-hydroxyanisole. Values expressed herein are mean ± SEM of three parallel measurements. *p* < 0.05. NA: not active.

**Table 6:** Enzyme inhibition activities of *T. nabateorum* extracts ^a^.

Sample	Anticholinesterase Inhibitory Activity		Tyrosinase Inhibitory Activity (Inhibition % at 100 µg/mL)	Urease Inhibitory Activity (Inhibition % at 50 µg/mL)	Antidiabetic Activity (IC_50_ µg/mL)	
	AChE (IC_50_ µg/mL)	BChE (IC_50_ µg/mL)			α-Amylase Inhibitory Activity	α-Glucosidase Inhibitory Activity
Methanol extract	^NA^	12.81 ± 0.35	10.51 ± 0.15	20.81 ± 0.45	31.99 ± 0.17	11.97 ± 2.07
Hexane extract	122.00 ± 0.47	13.28 ± 0.31	17.23 ± 0.36	15.23 ± 0.33	397.45 ± 0.23	NA
Essential oil	68.90 ± 3.07	14.26 ± 0.32	18.13 ± 0.31	16.88 ± 0.41	NA	NA
Galantamin ^b^	4.88 ± 0.61	46.88 ± 0.61	NT	NT	NT	NT
Kojic acid ^b^	NT	NT	74.12 ± 1.33	NT	NT	NT
l-mimosine ^b^	NT	NT	77.55 ± 1.21	NT	NT	NT
Acarbose ^b^	NT	NT	NT	NT	77.45 ± 0.98	190.23 ± 0.71
Thiourea	NT	NT	NT	73.08 ± 1.19	NT	NT

^a^ Values expressed herein are mean ± SEM of three parallel measurements. *p* < 0.05. NT: not tested. ^NA^: not active. ^b^ Reference compounds.

**Table 7:** EC50 values of *T. nabateorum* extracts on various cancer-cell lines.

	TN-Hexane (EC_50_)	TN-Methanol (EC_50_)
MDA-MB231	21.20 ± 2.09	53.88 ± 0.24
Caco-2	53.84 ± 1.38	101.91 ± 8.80
HepG2	38.00 ± 3.71	163.43 ± 4.20
LNCaP	19.60 ± 3.74	58.72 ± 3.00
HUVEC	190.79 ± 0.50	-

The values are means of three parallel measurements given as mean ± S.E.M. *p* > 0.05.

**Table 8:** Operating conditions of the ICP-MS instrument.

Instrument	Agilent™ 7700× ICP-MS
RF power	1600 W
RF match	2.10 V
Depth of sampling	10.0 nm
Nebulizer gas	0.57 L/min
S/C temperature	2 °C
Type of nebulizer	MicroMist
Spray chamber	Scott-type double-pass
Rate of flow	Plasma: 15 L/min. Auxiliary: 0.9 L/min. Nebulizer: 1.0–1.1 L/min
Rate of solution uptake	1.8 mL/min
Vacuum interface	4 torr, quadrupole: 2 105 torr
Acquiring data	Peak hopping. Replicate time 200 ms. Dwell time 200 ms. Sweeps/reading 3. Readings/replicate 3. Number of replicates 3

## Data Availability

Data will be made available on request.
